# Effect of complementary feeding behavior change communication delivered through community-level actors on the time of initiation of complementary foods in rural communities of West Gojjam zone, Northwest Ethiopia: a cluster-randomized controlled trial

**DOI:** 10.1186/s12887-020-02396-z

**Published:** 2020-11-05

**Authors:** Chalachew Abiyu, Tefera Belachew

**Affiliations:** Faculty of Public Health, Department of Nutrition and Dietetics, Jimma University, Jimma, Ethiopia

**Keywords:** Complementary feeding, Behavior change communication, Time of initiation of complementary food

## Abstract

**Background:**

Attaining the recommended level of complementary feeding practices remains a serious challenge in many developing countries. Complementary foods are usually untimely initiated, which has adverse consequences on the growth, development, and survival of infants. The focus of most studies conducted worldwide seemed to be on the effect of behavior change interventions on the adequacy of complementary diets; but not on the timing of initiations. Moreover, many of the interventions targeted only mothers/caregivers of infants, and studies that engaged the family members are scarce. This study aimed to evaluate the effectiveness of complementary feeding behavior change communication delivered through women development army leaderson the time of initiation of complementary foods.

**Methods:**

We conducted a cluster-randomized controlled trial in rural communities of West Gojjam Zone, Northwest Ethiopia from February 2017 to March 2018. A total of 16 geographic clusters were selected. Trial participants in the intervention group received complementary feeding behavior change intervention for 9 months whereas those in the control group received only the usual health care. Trained women development army leaders delivered the intervention. A pre-tested, structured interviewer-administered questionnaire was used for data collection. Generalized estimated equation (GEE) regression and survival analyses were used to test differences in time of initiation of complementary food between the study groups.

**Results:**

The intervention significantly improved the likelihood of timely initiation of complementary food by 22 percentage points [RR: 2.6; 95% CI: 1.78–5.86], and reduced the risk of late initiations by 19 percentage points [RR: 2.8; 95% CI: 1.83–4.37]. The complementary food initiation survival curve for the control group after 6 months was constantly above the curve than for the intervention group. The median age at the introduction of complementary food for infants was 6 months in the intervention group, and 6.7 months in the control group and the difference was statistically significant (*P*-value < 0.001).

**Conclusions:**

Complementary feeding behavior change communication improved the rate of timely initiation of complementary foods and reduced the risk of late initiations.

**Trial registration:**

ClinicalTrials.gov, NCT03488680. Registered 5 April 2018-*Retrospectively registered*, *https://clinicaltrials.gov/ct2/show/NCT03488680**.*

## Background

From all known health and nutrition preventive intervention strategies, optimal infant and young child feeding (IYCF) has the best significant impact on child growth and survival [1]. Optimal breastfeeding and complementary feeding practice prevent 13 and 6% of the deaths happening in under five children. Nonetheless, suboptimal feeding practices account for more than half of the deaths of under five children worldwide. Over two-thirds of these deaths are related to inappropriate feeding practices during the first 2 years of life [2].

The complementary feeding period is a critical time of transition in infants characterized by a gradual shift from breast milk to family food. The incidence of growth faltering increases significantly at 6 months of age when complementary foods are being introduced [3]. WHO recommends that mothers should initiate nutritionally adequate, safe, age-appropriate complementary foods at the sixth month of age (180 completed days), maintaining breastfeeding until the age of two years and beyond [4].

Attaining the recommended level of complementary feeding practices remains a serious challenge in developing countries including Ethiopia [5]. The rate of timely initiation of complementary food is lower than the WHO recommendation [6]. Complementary foods are initiated untimely (either too early or too late), which has adverse consequences on the growth, development, and survival of infants. Early initiation of complementary feeds (before the age of the sixth month) can lead to the displacement of breast milk and increased risk of infections, which further contributes to weight loss and malnutrition. Conversely, late initiation of complementary foods can result nutritional deficiencies and therefore as a consequence of malnutrition, the child is susceptible to morbidity [7].

The Ethiopian government carried out several efforts to enhance complementary feeding practices at different times through the implementation of IYCF guideline across the country [8]. However, these efforts failed to improve feeding practices at the expected level. The optimal complementary feeding practice was only 5%, and the timely initiation of complementary was 56% [9].

The promotion of optimal complementary feeding through behavior change interventions is a global health priority. The focus of most studies conducted worldwide seemed to be on the effect of behavior change interventions on the adequacy of complementary diets; but not on the timing of initiations. Moreover, many of the interventions targeted only mothers/caregivers of infants, and studies that engaged the family members are scarce [10].

In Ethiopia, a few behavior change interventions aimed at improving the IYCF practices have been conducted by Non-governmental organizations projects [11–14]. The reports of these projects focus either on implementation fidelity [11] or are implementation research [12], and large scale in scope [13, 15]. Moreover, none of the interventions targeted on age-specific complementary feeding practices, and delivered before infants entered the complementary feeding period (before 6 months). None of the projects also included control groups except a trial conducted in Hula woreda, Southern Ethiopia [14].

This trial aimed to evaluate the effectiveness of complementary feeding behavior change communication delivered through women development army leaders on the time of initiation of complementary foods. We hypothesized that the intervention is superior to the usual health and nutrition care in improving the timely initiation of complementary food and reducing the risk of early and late initiations. This study was part of a larger study entitled “effectiveness of complementary feeding behavior change communication delivered through community-level actors in improving feeding practices of mothers, health, and growth of infants in West Gojjam Zone, Northwest Ethiopia.” The study was conducted and the manuscripts are being prepared as part of the Doctoral dissertation. The main objectives of the study were to evaluate the effect of the intervention on the time of initiation of complementary foods, dietary adequacy, and linear growth of infants.

## Methods

### Study setting

The study was carried out in rural communities of West Gojjam Zone, Northwest Ethiopia from February 2017 to March 2018. West Gojjam Zone is one of the 13 administrative zones of the Amhara regional state. It has 13 rural districts*,* and each district is divided into *kebeles;* the lowest administrative units in Ethiopia*.* The zone has a total population of 2,560,131in 2016; of whom 1,262,144 were male and 1,297,987 were female. The rural part accounts for 92% of the total population. A total of 480,255 households were counted in this Zone, which results in an average of 4.39 persons to a household, and 466,491 housing units. From the total population mentioned, 315,228 were children of under five years of age of whom 160, 214 were under two years of age [16].

A total of 147,428 women Development Army (WDA) groups and 732,259 one-to-five networks were established in Amhara region [17]. The one-to-five networks are women volunteers who are empowered as WDA to transform their society. They are trained to focus more intensively on sparking local behavior change making regular rounds to check on neighbors and encourage healthy lifestyles. They are from “model families” and serve as living examples that the health extension workers (HEW) messages are being heard [18]. The proportion of women of childbearing age is 24% [19].

### The context

WDAs leaders are selected from the model families. A household that implemented all of the government’s 16 priority health interventions, from vaccinating their children and sleeping under mosquito bed-nets to building separate latrines and using family planning, is recognized as a model family [20]. “Model families” are selected by HEW in collaboration with the *kebele* administration [21]. WDAs leaders are unpaid health volunteers that undertake various preventive and promotive health services supported and supervised by HEWs [22].

Once the WDA groups are formed through community involvement, the WDA leaders provided 7 to 10 days training [19], to educate and mobilize the communities to utilize the maternal, neonatal and child health services [23]. On average, there are 30 WDA team leaders and 200 WDA network leaders in each *kebele* [19].

A WDA group consisted 25–30 households that are further organized into the “1 to 5” network of women where a model woman leads five other women within her neighborhood [24]. The one-to-five network functions as a forum for the exchange of concerns, priorities, problems, and decisions related to the health status of women. While being supported by the HEWs, the networks are responsible for the preparation of plans and ensuring their completion, for the collection of health information, and also for conducting a weekly meeting to review progress and submitting monthly reports [25]. The WDA groups thus support the implementation of the HEP.

**Fig. 1:**
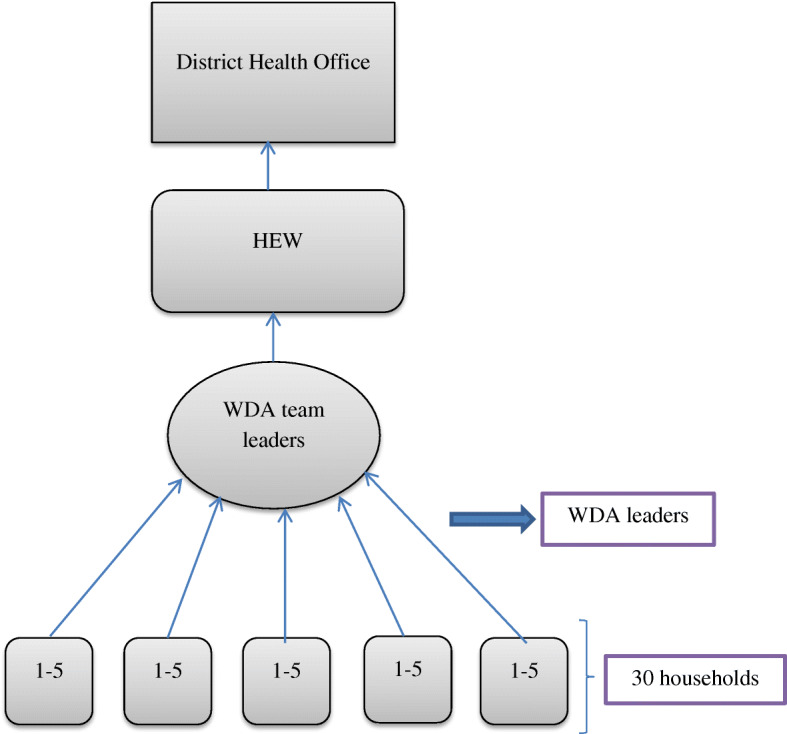
Hierarchy of WDA and reporting

The one-to-five networks meet every week, while the larger women development team meets once every two weeks. Moreover, they review their performance against their plan and evaluate each other monthly and give grades based on their performances. A performance report including the grades is organized at the health development team level and sent to the HEWs [19]. [Fig. 1].

In our study context, community-level actors are those people living in the community who could influence change in harmful traditional infant feeding behaviors and provide a supportive environment for the adoption of the recommended feeding practices. These include WDA leaders and the family members of the trial participants.

### Study design and population

A cluster-randomized controlled trial single-blind parallel-group, two-arm trials with a 1:1 allocation ratio was conducted among mothers of infants aged < 6 months of age at the time of enrolment. The trial was conducted in accordance with the CONSORT recommendations for cluster-randomized trials [26]. The intervention was delivered in community settings that encourage group participation. Hence, the unit of randomization was clusters (*kebeles)* to minimize intervention contamination and for logistical convenience. Consented mothers who were residents in the study area for at least 6 months before the start of the study were recruited and those who were ill and unable to communicate during the study were excluded.

### Sample size determination

In accordance with the main objectives of the larger study, the sample size estimates were designed to detect changes in the: 1) rate of timely initiation of complementary feeding 2) dietary diversity 3) linear growth. The sample sizes were calculated using *G-power* based on the following assumptions. Tail (s): One; α error probability = 0.05; power = 0.8; allocation ratio (N1/N2) = 1. Based on these assumptions, we arrived at the following sample size estimates: 1) 76 per group to detect a 20-percentage point anticipated difference in the timely initiation of complementary feeding (an increase from 46 to 66%). 2) 60 per group to detect a 15-percentage point anticipated difference in the minimum dietary diversity (an increase from 5 to 25%). These estimates were based on the baseline feeding practices reported in the Ethiopia Demographic and Health Survey (EDHS) [27]. 3) 139 per group to detect a 0.3 cm anticipated mean difference in linear growth (length gain) based on a systematic review of complementary feeding education interventions in developing countries that found a mean effect size of 0.21 cm (range 0.01–0.41 cm) for length gain [28].

The sample size was estimated based on linear growth since it gave the maximum sample size (*n* = 139 per group). To account for cluster design, it was multiplied by design effect of 2 and allowing for a 10% loss to follow up; the final sample size was 306 per group (a total of 612 in both study groups).

### Sampling and randomization

**Fig. 2:**
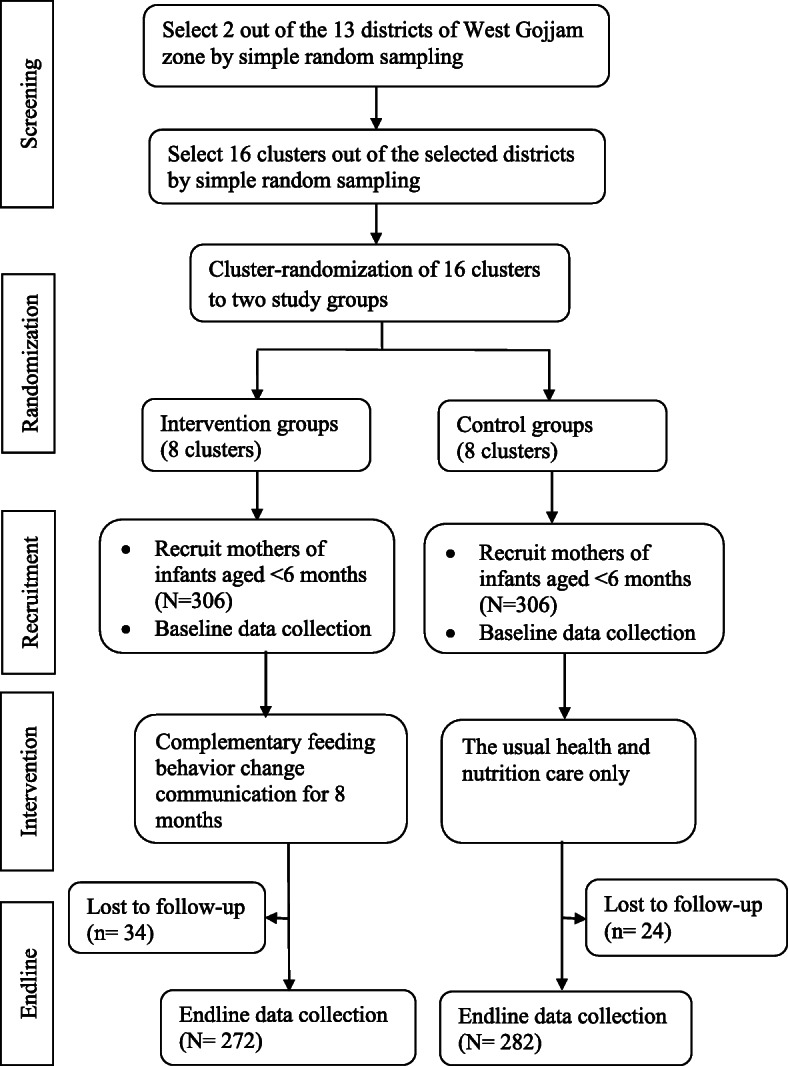
Trial profile

A two-stage cluster sampling technique was implemented. First, 2 out of the 13 districts in West Gojjam zone were selected by a lottery method. Second, lists of all clusters in the selected districts were compiled from the district administrative offices. The number of study subjects in the selected clusters was obtained from the records of births prepared by HEW. Each cluster in the selected districts forms the sampling frame; while the mother-infant pairs within the cluster formed the final sampling units of observation. Simple randomization with a 1:1 allocation was applied to assign clusters to either control or intervention groups. First, 16 non-adjacent clusters were selected by a lottery method. Then, the 16 clusters were listed alphabetically. A list of random numbers was generated in MS Excel 2010 and the generated values were fixed by copying them as “values” next to the alphabetic list of the clusters. These were arranged in ascending order according to the generated random number. Finally, the first 8 clusters were selected as intervention clusters and the last 8 as control clusters. A statistician that was blinded to study groups and not participated in the trial did the generation of the allocation sequence and the randomization of clusters. [Fig. 2].

### The intervention

The trial participants in the intervention group delivered complementary feeding behavior change communication for 9 months whereas those in the control group delivered only the usual care. The language of communication during the intervention period was Amharic (the local language). The intervention had three parts.

### Part 1: training of women development army (WDA) leaders

A total of 24 WDA leaders were recruited by HEWs in the intervention clusters (3 in each cluster) and centrally trained by the researcher. The training focused on the key messages on optimal complementary feeding practices followed by cooking demonstrations. The first training session was conducted at the beginning of the intervention whereas the second session of similar content was repeated at the middle (4 months) of the intervention period and each session lasted for 3 days. After training sessions, every participant received a copy of the visual materials (posters) containing the key messages for complementary feeding practices to be used as a reference.

The training contents were adopted from the Alive and Thrive program in Ethiopia. Key messages were compiled into posters used to deliver training [29]. Direct, interactive and participatory learner and activity-oriented instructional strategies were delivered. Talks, group discussions, group work exercises, demonstrations, role plays, storytelling, simulation, case studies and problem-solving were used to enhance knowledge, attitude, and behaviors. The intervention key messages were focused on the correct time to initiate complementary foods; specific foods to be given or avoided and how to give them; meal frequencies; amounts of foods to be fed to infants at different ages while continuing breastfeeding; offering a variety of foods from different food groups; practice responsive feeding; practice good hygiene, and continue to feed the child during and after an illness. [Table 1].

**Table 1:** Complementary feeding practices key messages in the intervention clusters

No.	Key messages
1	Start feeding your baby soft and thick porridge made from a combination of cereal flours at 6 months. Continue to breastfeed your child up to 2 years and beyond.
2	Enrich baby’s porridge by adding one or more ingredients from animal-source foods (milk, egg, dried meat powder), finely chopped vegetables (kale, carrot, cabbage, tomato, potato) and mashed fruits (avocado, papaya, mango, banana, pumpkin) in each meal.
3	Cook and feed animal-source foods (e.g. eggs, beef, pork, chicken, liver, fish) at least 3 times per week. Feed your child a fruit (e.g. ripe banana, mango, orange, papaya, avocado) after a meal at least once per day.
4	Increase variety, amount and frequency of feeding with age for the baby. Amount of food per meal: Begin with 2 to 3 tablespoons at 6 months of age. 2 to 3 tablespoonfuls; and increase gradually to half (½) 250 ml cup at 6–9 months. Half (½) of 250 ml cup at 9–12 months. Three-quarters (¾) to 1 of 250 ml cup at 12–24 months. Frequency of feeding per day: 2–3 times at 6–8 months, 3–4 times at 9–23 months. Feed 1–2 snacks (e.g. sliced bread, fruits) between two major meals.
5	Encourage your child to eat with patience and love. Interact and minimize distractions during feeding. Don’t force your baby to eat. Provide extra food during and after an illness.
6	Feed your baby using a clean cup and spoon; avoid bottle feeding. Wash your hands with soap and water before preparing food, before eating, and before feeding young children.
7	Enriched baby’s porridge preparation: •Prepare a germinated flour made up of 3/4th staples (one or more ingredients from maize, wheat, rice, millet, sorghum, oat) and 1/4th legumes (one or more ingredients from beans, lentils, chickpeas, groundnuts). •Use milk instead of water for preparing porridge. •Add butter/oil which will make the thick porridge easier to eat. •Add finely chopped meat, fish or eggs. •Add one or more ingredients from finely chopped vegetables and mashed fruits. •Increase the consistency and thickness of the porridge with child age. •Do not forget to use iodized salt.

### Part 2: group training of mothers by WDA leaders

Each WDA leader was assigned to 10–15 mothers with children younger than 6 months residing in their village. They delivered a total of nine group training sessions including cooking demonstrations (once per month, for 3 days duration each) for the mothers they are assigned with the same training procedures provided by the researcher in part 1. WDA leaders culturally appropriate training sessions with mothers using posters prepared in the local language.

### Part 3: home visits

A total of nine home visits (once per month, for 2 days duration each) were delivered by each WDA leader in the intervention clusters that aimed to bring behavior change at maternal and family level. During each home visit, individual counseling and support were deliver for each mother to facilitate the implementation the optimal of feeding practices she had been taught during the training sessions, to observe feeding practices, to demonstrate cooking procedures, to correct the harmful practices and to provide feedback focusing on the optimal complementary practices. A participatory discussion was held with family members (fathers and grandmothers of the recruited infant) regarding optimal complementary feeding practice, its impact on children’s nutrition and health; and how can they support the mother in feeding the baby. Each mother provided a poster containing the key messages at the end of each home visit.

All the activities of WDA leaders were supervised and monitored by HEWs and the overall supervision and monitoring were done by the researcher. All activities done during the study period are presented in [Table 2]. Recruitment of study participants and baseline data collection was conducted between February and March 2017. Following the baseline survey, the intervention was delivered for the intervention clusters from April 2017 to December 2017. The endline data collection was carried out between January and February 2018.

**Table 2:** Schedule of activities during the study period

Activities	Time points in months												
	1	2	3	4	5	6	7	8	9	10	11	12	13
Enrollment and baseline data collection	x^**I + C**^	x^**I + C**^											
Training of WDA leaders			x^**I**^				x^**I**^						
Group training of mothers			x^**I**^	x^**I**^	x^**I**^	x^**I**^	x^**I**^	x^**I**^	x^**I**^	x^**I**^	x^**I**^		
Home visits			x^**I**^	x^**I**^	x^**I**^	x^**I**^	x^**I**^	x^**I**^	x^**I**^	x^**I**^	x^**I**^		
Process evaluation			x^**I**^	x^**I**^	x^**I**^	x^**I**^	x^**I**^	x^**I**^	x^**I**^	x^**I**^	x^**I**^		
Endline data collection												x^**I + C**^	x^**I + C**^
Supervision	x^**I + C**^	x^**I + C**^	x^**I**^	x^**I**^	x^**I**^	x^**I**^	x^**I**^	x^**I**^	x^**I**^	x^**I**^	x^**I**^	x^**I + C**^	x^**I + C**^

^**I**^Intervention groups; ^**C**^Control groups; ^**I + C**^Activities both in intervention and control groups

### Blinding

Data collectors were not informed of the allocation clusters and were not residents in any of the clusters. However, trial participants knew the intervention allocation due to the nature of the intervention.

### Process evaluation

Process evaluation was conducted to document the intervention implementation process and assess whether the intervention activities were implemented as planned, evaluate the performance of WDA leaders and the extent to which the intervention reached the intended mothers and family members. [Table 3].

**Table 3:** Process evaluation

Data sources	Process indicators	Characteristics
**1. Assess whether the intervention activities are implemented as planned**		
Activity logs	•Number of training sessions including cooking demonstrations held with WDA leaders •Number of visual materials distributed to WDA leaders •Number of training sessions including cooking demonstration held with mothers •Number of visual materials distributed to mothers	Fidelity
**2. Evaluate the performance of WDA leaders**		
Attendance records	•Number of recruited WDA leaders •Number of WDA leaders trained •Number of home visits conducted by WDA leaders	Dose delivered (exposure)
**3. Evaluate the extent to which the intervention reached the intended mothers and family members**		
Attendance records	•Number of recruited mother-infant pairs •Number of mothers trained •Number of mothers attended home visits •Number of family members attended home visits	Dose delivered (exposure)

### Data collection methods and outcome measurements

A baseline survey was conducted following enrolment of mothers using a pre-tested structured interviewer-administered questionnaire to assess child, maternal and household characteristics in both study groups at the same time. At the end of the intervention period, an endline data collection was carried out to examine the time of complementary food initiation to children in both the intervention and control groups at the same time. Recruited infants aged < 6 months at baseline survey achieved 8–14 months of age at the time of endline data collection.

The time of complementary food initiation was assessed according to the key indicators recommended by WHO [30]. Accordingly, the outcome variables (early, timely and late initiation of complementary food) were determined by asking the mothers to recall the age at which they first initiated any solid, semi-solid, or soft foods to the index child in addition to breast milk. If the mother initiated complementary food for the child before the sixth month (before 180 completed days), it was categorized as “early initiation of complementary food”; if she had initiated exactly at the sixth month (180 completed days), it was categorized as “timely initiation of complementary food” and if she had initiated after the sixth month (after 180 completed days), it was categorized as “late initiation of complementary food”. For a few mothers who encountered difficulties in remembering the right age they introduced complementary food for their children, data collectors conducted different probing techniques to help them recall, thereby recall bias was minimized. Some of the probing techniques were relating the time of initiation to known public events, occurrences of common childhood developmental milestones, and immunization schedules. Moreover, data collectors arranged a comfortable environment by keeping mothers apart and making them free during data collection to minimize social desirability bias.

### Data quality control

The questionnaire was prepared in English, and translated to the local language Amharic and then back to English by experts of the language to keep its consistency. Training on data collection tools was given to data collectors and supervisors. A pre-test was done on 5% of the sample to assess the clarity of the questions and applicability of the instrument. To enhance blinding, precise objectives of the study and cluster allocation to the trial were not disclosed to data collectors and the data collection schedule was randomized. WDA leaders were not involved in data collection. Daily supervision was conducted by the supervisors and principal investigator.

### Data processing and analyses

Data were double entered into the EPI-Info, exported to SPSS version 21 for cleaning and statistical analysis. Baseline characteristics of the study groups were presented using descriptive statistics. Generalized estimated equations (GEE) regression analyses adjusted for clustering were used to test the effect of the intervention on the time of initiation of complementary food. Relative risks (RR) and risk differences (RD) with 95% confidence interval (CI) were computed as outcome a measure of the intervention effect. Life table survival analysis was conducted to estimate the likelihood of complementary food initiation to children at each month between the study groups. Kaplan-Meier survival analysis was done to examine the time to initiation of complementary food. The long rank test was used to assess the presence of significant differences between the control and intervention groups. All analyses were conducted according to the intention to treat (ITT) principle and the adjusted effect measures were considered as the main results. *P*-value < 0.05 was considered as statistically significant.

### Ethical standards disclosure

All procedures involving the research were approved by Jimma University College of Health sciences institutional and review board. Permission to undertake the study was obtained from the regional, zonal and district administration and health offices of the study area. After the identification of eligible mothers, the nature and purpose of the study were explained along with their right to refuse. Written informed consent was obtained from all study participants. The right of the participant to withdraw from the study at any time was respected. The data were not accessed by a third person, except investigators, and were kept confidential. The study was registered at clinicaltrials.gov as NCT03488680 in April 2018. The trial was registered after the enrolment of the study participants because the registration process taken unpredictably long time than we expected. The enrolment of the trial participants began before the trial was registered to conduct the trial according to the time plan. The authors confirm that all ongoing and related trials for this intervention are registered.

## Results

At baseline, a total of 612 mother-child pairs (306 in the control and 306 in the intervention group) were recruited yielding a response rate of 100%. Of these, 34 (11%) in the intervention group and 24 (7.8%) in the control group were excluded in the study because they moved away from the study area, decided not to continue in the study or were lost to follow-up during the endline data collection. Overall, endline data were completed for 554 (90.5%) of the study participants in both study groups.

### Baseline characteristics

Baseline infant, maternal, and household characteristics of the study groups is presented in [Table 4].

**Table 4:** Baseline characteristics of the study participants

Variable	Control group (***N*** = 282)	Intervention group (***N*** = 272)
**Child**		
Sex (%)		
Male	55.3	54.6
Female	44.7	45.6
Age (months), mean + SD	3.22 + 1.4	3.21 + 1.48
**Maternal**		
Age (months), mean + SD	27.2 + 5	28.05 + 4.8
Educational status (%)		
Attended formal education	23.8	19.4
No formal education	76.2	80.6
Occupation (%)		
Farmer	12.1	10.8
Housewife	87.9	89.2
Marital status (%)		
Single	2.1	1.8
Married	93.6	94.6
Divorced	3.1	2.6
Widowed	1.1	1
Parity (%)		
Primiparous	16.7	19.8
Multiparous	83.3	80.2
Perception of child’s weight (%)		
Large	26.6	28.9
Medium	48.6	45.6
Small	24.5	25.3
ANC visit (%)		
Yes	73.4	71.4
No	26.6	28.6
Place of delivery (%)		
Home	63.5	64.8
Health facility	36.5	35.2
PNC checkup (%)		
Yes	27	22.7
No	73	77.3
IYCF counseling (%)		
Yes	33	30.4
No	67	69.6
Knowledge score on CFP, mean + SD	6.35 + 1.86	6.33 + 1.66
Attitude score on CFP, mean + SD	5.55 + 2	5.76 + 1.65
**Household**		
Family size, mean + SD	5.5 + 1.8	5.3 + 1.9
Possession of radio (%)		
Yes	19.5	22
No	80.5	78
Listens to *seven solutions* (%)		
Yes	27.3	21.7
No	72.7	78.3

*CFP complementary feeding practices

*Seven solutions: a radio drama focused on infant and young child feeding (IYCF) practices

### Effects of the intervention

Binary generalized estimated equation (GEE) regression analyses were done to test the effects of the intervention on the time of complementary food initiation for children between the control and intervention groups. Complementary food was initiated for 264 (97%) children in the intervention group and 258 (91.5%) in the control group at the time of data collection. The proportions of early, timely, and late initiations of complementary food were compared among mothers who initiated complementary foods for their children between the study groups. The proportion of early initiation of complementary food was higher in the control, 28 (11%), than intervention group, 22 (8%), but the difference was not statistically significant [RR = 1.3; 95% CI: 0.75–3.86]. On the other hand, there was a statistically significant difference in the proportion of timely initiation of complementary food which was higher in the intervention, 206 (78%) as compared to the control group, 145 (56%) [RR: 2.6; 95% CI: 1.78–5.86]. The proportion of late initiation of complementary food was higher in the control, 83 (33%), than the intervention group, 36 (14%), and the difference was statistically significant [RR: 2.8; 95% CI: 1.83–4.37]. [Table 5].

**Table 5:** Generalized estimated equation regression analyses on the time of initiation of complementary food for infants by study groups

Variable	Study group	***N*** (%)	RR (95% CI)	RD (95%CI)
Early initiation	CG	28 (11)	1.3 (0.745–3.857)	0.03 (−0.824–1.126)
	IG	22 (8)	1	0
Timely initiation	CG	145 (56)	1	0
	IG	206 (78)	2.6 (1.778–5.862)	2.3 (1.418–6.457)
Late initiation	CG	85 (33)	2.8 (1.825–4.370)	0.4 (1.256–3.714)
	IG	36 (14)	1	0

*CG* control group, *IG* intervention group, *RR* relative risk, *RD* Risk difference, *CI* confidence interval

**Fig. 3:**
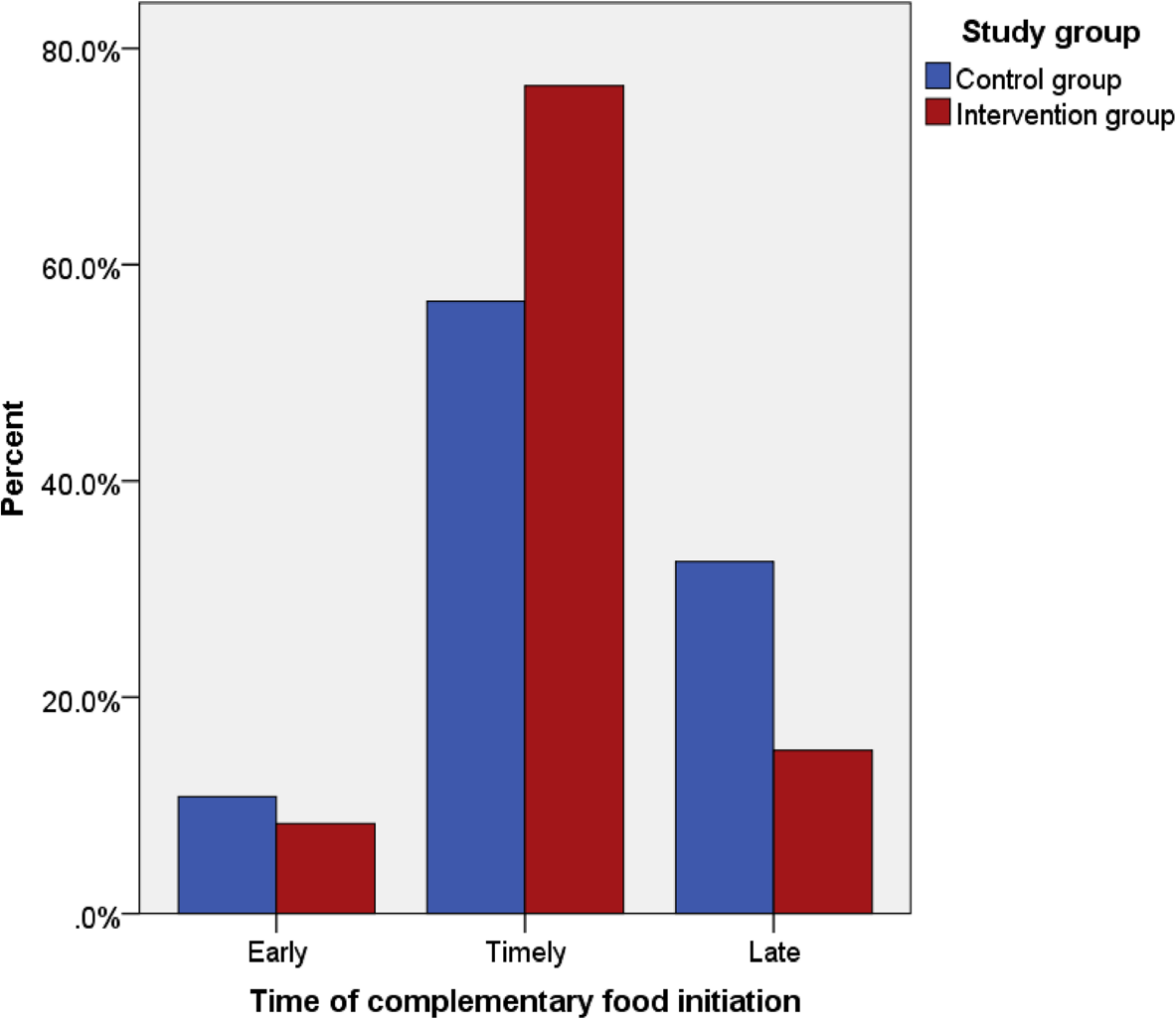
Proportion of time of initiation of complementary foods by study groups

The proportion of time of initiation of complementary food between the control and intervention groups is also presented in Fig. 3. The proportions of early, timely, and late initiations were 8, 78 and 14% in the intervention group, whereas it was 11, 56 and 33% in the control group, respectively. [Fig. 3].

Table 6 shows the cumulative survival probabilities of complementary food initiation at different ages of children for control and intervention groups. The life table, for example, indicated that the cumulative survival probability of complementary food initiation for the first 6 months was higher in the control group, 37%, than the intervention group, 17%. This showed that a higher proportion of children in the control group as compared to those in the intervention group did not receive complementary food for the first 6 months. Likewise, complementary food was not introduced for 14% of children for the first 8 months in the control group, which was only 3% in the intervention group.

**Table 6:** Cumulative survival probabilities and hazard rates of complementary food initiation at different ages of infants in months between the study groups

Study group	Time of complementary food initiation (months)	Interval start time (months)	No. of cases entering this interval	No. of censored cases	No. of terminal events	Proportion of surviving	Cumulative survival probability at the end of interval	Hazard rate
**Control Group**	0–1	0	282	0	0	1.00	1.00	.00
	1–2	1	282	0	0	1.00	1.00	.00
	2–3	2	282	0	3	.99	.99	.01
	3–4	3	279	1	5	.98	.97	.02
	4–5	4	273	0	2	.99	.96	.01
	5–6	5	271	2	18	.93	.90	.07
	6–7	6	251	3	146	.41	.37	.83
	7–8	7	102	6	31	.69	.26	.37
	8–9	8	65	6	27	.56	.14	.56
	9–10	9	32	6	24	.17	.02	1.41
	10–11	10	2	0	2	.00	.00	.00
**Intervention group**	0–1	0	272	0	0	1.00	1.00	.00
	1–2	1	272	0	0	1.00	1.00	.00
	2–3	2	272	1	0	1.00	1.00	.00
	3–4	3	271	0	4	.99	.99	.01
	4–5	4	267	0	4	.99	.97	.02
	5–6	5	263	1	14	.95	.92	.05
	6–7	6	248	2	202	.18	.17	1.38
	7–8	7	44	0	24	.45	.08	.75
	8–9	8	20	4	12	.33	.03	1.00
	9–10	9	4	0	4	.00	.00	2.00

**Fig. 4:**
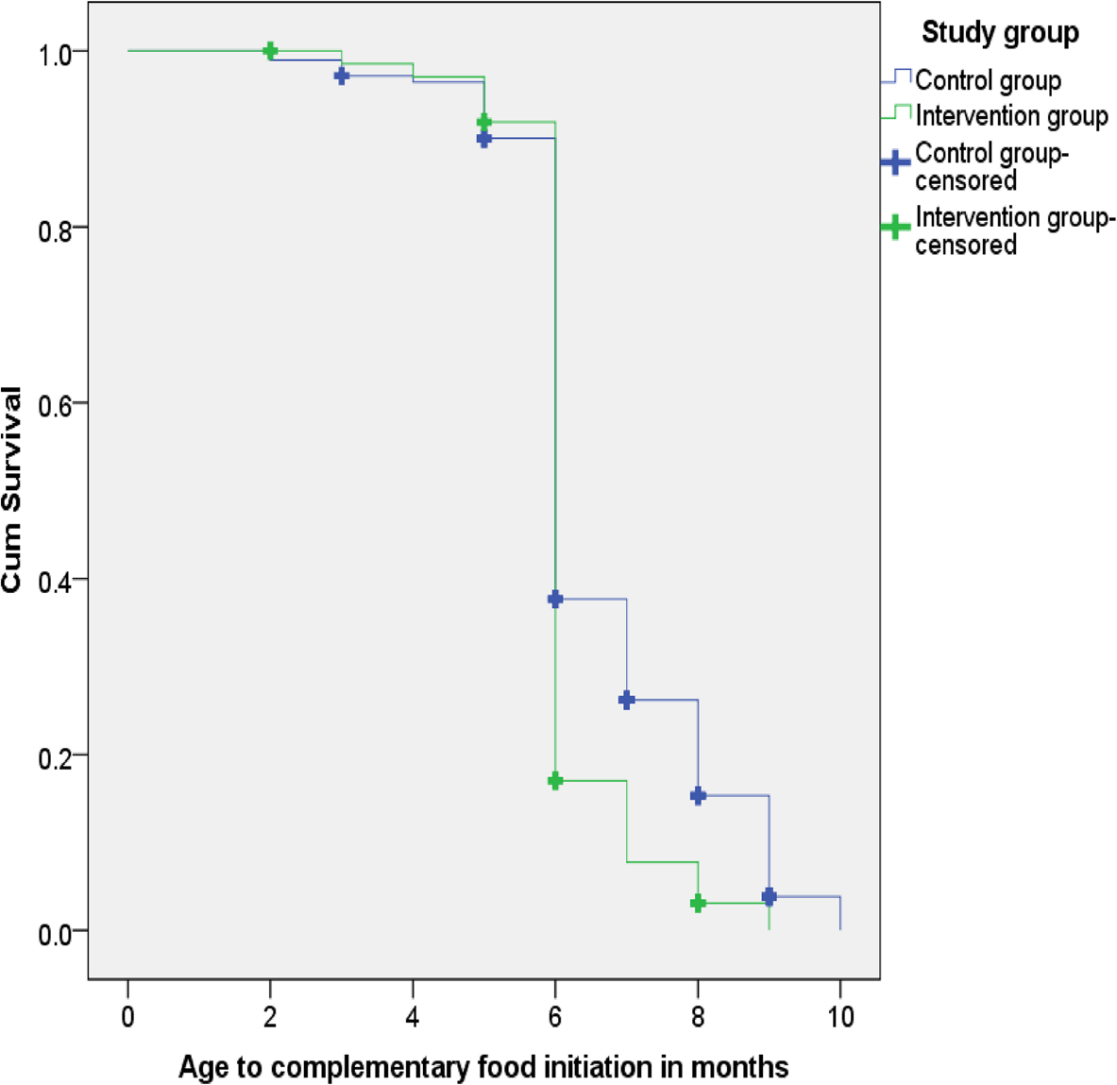
Kaplan-Meier curve on time to initiation of complementary food by study groups

Kaplan-Meir survival analysis on Figure-4 shows that the time to initiation of complementary food for the control group after 6 months was constantly above the curve for the intervention group. The difference was statistically significant on a Log Rank (Mantel-Cox) test (*p*-value < 0.001). The median age for initiation of complementary food was estimated 6.7 months for control groups and 6 months for the intervention groups. [Fig. 4].

## Discussion

WHO recommends that mothers should initiate nutritionally adequate, safe, age-appropriate complementary foods for children at the sixth month of age, maintaining breastfeeding until the age of two years and beyond. Nonetheless, the rate of timely initiation of complementary food is very low in developing countries including Ethiopia. Complementary foods are initiated untimely; either too early or too late [4]. Therefore, it is crucial to design intervention strategies that can improve the timely initiation of complementary food. This study investigated the effect of complementary feeding behavior change communication on the time of initiation of complementary food.

All mothers were asked to note down the exact age at which they first introduced complementary food for their infants to determine the time of initiation of complementary foods. Most of the mothers reported the exact age they first introduced complementary foods. For a few mothers who encountered difficulties in remembering the right age, data collectors conducted different probing techniques to help them recall the exact age of initiation. Some of the probing techniques were relating the time of initiation to known public events, occurrences of common childhood developmental milestones, and immunization schedules.

The main findings of this study indicated that the intervention had statistically significant effects on the rate of timely and late initiation of complementary food. The likelihood of timely initiation of complementary food was 2.6 times more in the intervention group as compared to those in the control group [RR: 2.6; 95% CI: 1.78–5.86]. Conversely, mothers in the control group were 2.8 times more likely to initiate complementary food lately (after the age of six months) as compared to those in the intervention group [RR: 2.8; 95% CI: 1.83–4.37].

Our finding is in line with the results of a systematic review of four cluster-randomized trials [10]. In this review, the pooled effect estimate suggests that, compared to standard care, the educational intervention significantly reduced the risk of early introduction of complementary food (before four to six months of age) by 12 percentage points [average RR: 0.88; 95% CI: 0.83–0.94). Studies used intervention delivery strategies that ranged from counseling sessions to the use of printed materials, flip charts and videos, with some studies using a combination of at least two of the listed delivery strategies.

A cluster randomized controlled trial conducted in rural Cambodia found no significant differences in the proportions of children on which complementary food is introduced at the of 6–8 months between the intervention (88%) and control group (92.6%), (*P*-value: 0.349) [31]. Trained community nutrition promoters together with local NGO conducted seven nutrition education sessions for interested caregivers with children aged 5–18 months in the intervention villages. The insignificant effect of the intervention in the Cambodian study could be due to the rate of timely initiation of complementary food was already high at baseline in both control and intervention groups. There was also a difference in the definition of the right age for the introduction of complementary foods between the Cambodian study (at the age of 6–8 months), and our study. WHO recommends the right age for the introduction of complementary food for infants is at the age of the sixth month [4].

The life table survival probability distribution, in our study, indicated that a higher proportion of children did not receive complementary food for the first 6 months in the control group (37%) as compared to those in the intervention group (17%). Likewise, complementary food was not introduced for14% of children for the first 8 months in the control group, which was only 3% in the intervention group.

In our study, the Kaplan-Meir survival analysis showed that the survival curve for the control group after 6 months was constantly above the curve for the intervention group. The median age for initiation of complementary food for children was higher in the control group (6.7 months) as compared to those in the intervention group (6 months), and the difference was statistically significant (*P*-value < 0.001).

A similar result was found by a cluster-randomized controlled trial conducted in rural Brazil. In the Brazilian study, mothers and grandmothers in the intervention group received a total of five counseling sessions on breastfeeding and healthy complementary feeding at the maternity ward and home whereas the control group received the usual care. The median age at the introduction of complementary food was 5 months in the intervention group and 4 months in the control group and the difference was statistically significant (P-value < 0.01). The educational intervention improved the age at which complementary foods are introduced for infants [32].

In general, the focus of most of the studies conducted worldwide was to investigate the effect of behavior change interventions on the adequacy of complementary foods of children. Studies that evaluated the effect of such interventions on the time of initiation of complementary food are very limited worldwide. Nonetheless, the age at which complementary foods are introduced is an important factor for optimal growth and development of children. Both early and late initiations have adverse consequences on children’s health and nutrition.

Early initiation of complementary feeds can lead to the displacement of breast milk and increased risk of infections, which further contributes to weight loss and malnutrition. Conversely, late initiation of complementary food is also associated with negative consequences to the infant’s health. As breast milk is no longer able to sustain the nutritional requirements of an infant after six months of age, continuing to feed only breast milk beyond this period leads to nutritional deficiencies and therefore as a consequence of malnutrition, the child is susceptible to morbidity [33].

### Strength and limitation of the study

The strengths of this study are the use of the cluster-randomized design, and the intervention engaged not only mothers/caregivers but also family members. The intervention effects would be also sustainable because it was delivered in a community supportive environment. Our study has some limitations. First, due to the nature of the study, it was not possible to conduct a double-blind trial. So, mothers in the intervention group might provide the correct answer to the questions asked rather than to report the true practices. This will result in social desirability bias despite data collectors arranged a comfortable environment by keeping mothers apart and making them free during data collection. Second, the age to the introduction of complementary food was determined based on maternal recall of the age at which they first initiated complementary food. Hence, recall bias can not be excluded even if data collectors applied various probing techniques to help mothers remember the correct age.

## Conclusions

This study indicates the potential effectiveness of complementary feeding behavior change communication delivered through community-level actors in improving the rate of timely initiation of complementary foods and reducing the risk of late initiations. The result suggests also behavior change intervention that engaged not only mothers of infants but also their family members could be an effective approach.

## Data Availability

The datasets generated and/or analyzed during the current study are not publicly available due to confidentiality but are available from the corresponding author on reasonable request.

## Abbreviations

CI: Confidence interval
EDHS: Ethiopian demographic and health survey
HEP: Health extension program
HEW: Health extension worker
IYCF: Infant and young child feeding
RR: Relative risk
WDA: Women development army
WHO: World health organization
